# Performance Evaluation of Tunnel-Slag-Improved High Liquid Limit Soil in Subgrade: A Case Study

**DOI:** 10.3390/ma15051976

**Published:** 2022-03-07

**Authors:** Xiaomeng Zheng, Zhushan Shao, Nannan Zhao, Chenglong Li, Kui Wu

**Affiliations:** 1 School of Civil Engineering, Xi’an University of Architecture and Technology, Xi’an 710055, China; gequykigeqk@163.com (X.Z.); shaozhushan@xauat.edu.cn (Z.S.); zhaonannan@xauat.edu.cn (N.Z.); 2 School of Science, Xi’an University of Architecture and Technology, Xi’an 710055, China; 3 Shum Yip East China Real Estate Development Co., Ltd., Nanjing 210011, China; zhao0008@126.com

**Keywords:** tunnel slag, high liquid limit soil, mechanical property, feasibility, engineering application

## Abstract

The application of tunnel-slag-improved high liquid limit soil as filling materials in subgrade is a green environmental technology. This study explored the influence of tunnel slag mixing on the physical and mechanical properties of improved soils, based on the engineering background of Liyu highway, Guangxi Province, China. Firstly, the optimal moisture content, maximum dry density, shear strength parameters, California bearing ratio (CBR) and resilience modulus of plain and tunnel-slag-improved high liquid limit soils were experimentally determined. Results showed that the direct utilization of untreated soil was unacceptable in subgrade practice. A significant enhancement of integrity of high liquid limit soils could be obtained by tunnel slag mixing, and the value of 15% was determined as the optimal tunnel slag content in soils, leading to improved soil performance meeting the specification requirements. Then, numerical simulation on the stability of subgrade slope of tunnel-slag-improved soils at the content of 15% was conducted. It also reported the long-term subgrade settlements. The feasibility of utilization of tunnel slag in improving properties of high liquid limit soils was further validated. Finally, a good application of tunnel-slag-improved high liquid limit soil as subgrade filling materials in Liyu highway was achieved. The findings in this study could provide useful guidance for similar engineering.

## 1. Introduction

Soft soil deposits are prevalent in coastal areas, deltas and floodplains throughout the world [1]. However, it is infeasible to have a direct use of these soft soils in geotechnical practice because of their high moisture content and low bearing capacity [2,3,4,5]. In highway subgrade construction, the utilization of untreated soft soils as the filling materials may make it difficult to achieve the optimal moisture and required compaction degree after rolling, leading to a low bearing capacity, excessive settlement and possible slope instability under the traffic loadings. Therefore, it is very necessary and important to improve the properties of soft soils prior to employing them as subgrade filling materials.

Many researchers have attempted to use various methods in order to obtain soft soils with high performances. Mujah et al. [6] explored the possible reuse of ground palm oil fuel in soft soil improvement and found the shear strength parameters of the improved soil could increase remarkably by approximately 50–60%. Abukhettala and Fall [7] showed that the addition of plastic wastes decreased the maximum dry density and increased the CBR value of subgrade soils, making it possible to have a partial replacement of soils with plastic waste in improving the properties of subgrade soils. Chen and Lin [8] reported the possible application of incinerated sewage sludge ash mixed with cement in soft soil subgrade. Majumder and Venkatraman [9] discussed the influence of lime on the plasticity index and CBR value of black cotton soil. Negawo et al. [10] also showed a series of experimental results reporting the improvement in mechanical properties of lime-mixed soft clay soils. Du et al. [11] compared the improvement effects of calcium carbide residue and quicklime in soft soil subgrade and concluded that the utilization of calcium carbide residue could lead to a better performance. Yorulmaz et al. [12] experimentally found the inclusion of an appropriate amount of waste marble powder could positively improve the bearing capacity of the subgrade soils of highly plastic silt and highly plastic clay. Malicki et al. [13] presented the CBR test results of the effect of recycled geogrid on the bearing capacity of subgrade soft soils.

High liquid limit soil, as a type of soft soil, has often been encountered in highway subgrade construction in the southern mountains of China. It generally exhibits the characteristics of high moisture content, high porosity ratio, low permeability and high compressibility [14,15,16]. Numerous studies specifically focusing on the improvement of properties of high liquid limit soil have been carried out. Cheng et al. [17] reported the influence of sand mixing on the bearing capacity of high liquid limit soil and determined the optimal ratio of sand based on the Yunluo expressway, China. However, it should be accepted that the mass exploitation of river sand is required if sand-improved high liquid limit soil is applied as a subgrade filling material, causing great damage to the environment. Zeng et al. [18] experimentally found that the addition of lime in high liquid limit soil leads to a decrease in its liquid limit, plastic index and dilatability. Zhang et al. [19] investigated the relationship between liquid/plastic limit, free swell, CBR and soakage of high liquid limit soil and mix ratio of quicklime. Mo et al. [20] concluded that cement mixing could significantly decrease the hydrophilicity and increase the compaction degree and CBR value of high liquid limit soil. Unfortunately, it was reported that it would take a very long time for lime or cement to fully react in high liquid limit soil. In addition, lime and cement are relatively expensive, causing a high increase in construction cost. Kianimehr et al. [21] and Sharma and Sharma [22] reported a significant increase in the properties of improved high liquid limit soils using construction and demolition wastes and considered that recycling construction and demolition wastes into subgrade materials was a promising way to process these wastes. However, the improvement was not significant and the service life of improved soil was too short. In fact, a large amount of tunnel slag would be produced in mountain areas. It is still a challenging task for engineers to dispose of a huge amount of tunnel slag without environmental pollution [23,24,25]. Tunnel slag shows features similar to those of construction and demolition wastes, and it is considered to be acceptable to use the tunnel slag in the high liquid limit soil. This green environmental technology can both address the problem of disposal of tunnel slag and provide subgrade filling materials with higher performance, and it has been increasingly promoted in highway subgrade practice. However, the mechanical properties and stability mechanism of tunnel-slag-improved high liquid limit soil have not been comprehensively and systematically investigated.

Based on the engineering background of Liyu highway, Guangxi Province, China, this study discussed the feasibility of utilization of tunnel-slag-improved high liquid limit soils in subgrade. A series of laboratory tests on physical and mechanical properties of plain and improved high liquid limit soils were carried out, and the optimal content of tunnel slag mixed in soils was determined. The stability of the subgrade slope of tunnel-slag-improved soil and subgrade settlements were analyzed by using a numerical method. Finally, an attempt was made to apply tunnel-slag-improved high liquid limit soils in the subgrade practice of Liyu highway.

## 2. Engineering Background

**Figure 1:**
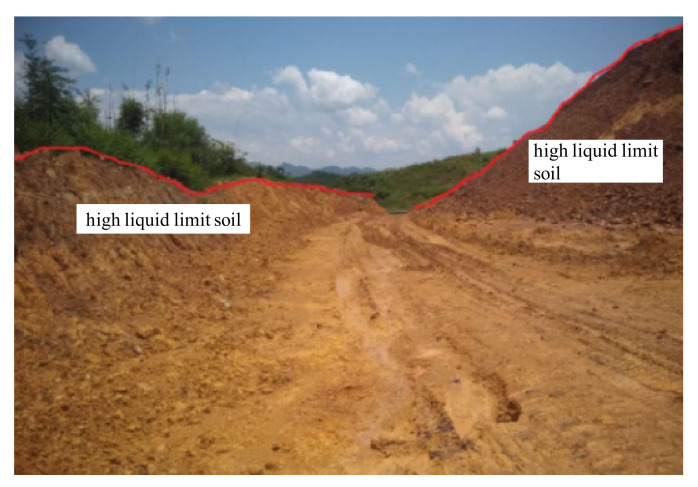
High liquid limit soil in Liyu highway.

The Liyu highway is located in Mengshan County, Wuzhou City, Guangxi Province, China [26,27]. The total length of this highway is 14.545 km, including nine bridges and a superlong tunnel (Wenwei tunnel). The subgrade length in Liyu highway accounts for 11.925 km, and the length of subgrade of high liquid limit soil is up to 3.25 km (see Figure 1), being approximately 27.25% of the total subgrade length. In this project, it was a very difficult problem for engineers to deal with such a mass of high liquid limit soil subgrade. Considering the huge amount of tunnel slag with high strength produced in the Wenwei tunnel, engineers attempted to utilize the tunnel slag in the improvement of properties of high liquid limit soils. It not only addressed the problem of high liquid limit soil subgrade but also provided a solution for the reuse of tunnel slag.

The Wenwei tunnel was designed as separate tunnels. The starting and ending points of the left tunnels are located at ZK61 + 625 and ZK66 + 295, respectively, with a total length of 4670 m. The right tunnel has a total length of 4677 m, with the starting and ending points of YK61 + 638 and ZK66 + 315, respectively. The maximum overburden depth of Wenwei tunnel is up to 368 m. The method of blasting excavation was used in Wenwei tunnel, making it possible to better use tunnel slag in improving subgrade soil performance. The tunnel slag taken from Wenwei tunnel is mainly composed of completely weathered gneiss, which has good mechanical properties. Based on this, the properties of plain and tunnel-slag-improved high liquid limit soil were investigated. The studied high liquid limit soil was taken from section K58 + 235~K58 + 400 of Liyu highway.

## 3. Physical and Mechanical Properties of High Liquid Limit Soil

### 3.1. Physical Properties of High Liquid Limit Soil

#### 3.1.1. Particle Analysis

The grain composition of soil determines its structural characteristics and physical and mechanical properties and is an important index for soil classification. Therefore, the particle analysis test of high liquid limit soils in Liyu highway was first conducted. It should be noted that, in this and following tests, the specifications of China were referenced, considering that the quality of Liyu highway should meet the Chinese standard. In spite of this, the study would provide a positive and feasible way to process high liquid limit soil subgrade for readers from different countries. According to the Test Method of Soils for Highway Engineering (JTG3430-2020) of China [28], 2000 g of soil sample was used in this particle analysis test. Two treatment processes of coarse screening and fine screening were applied for the soil sample. The specific steps were as follows: (1) stacking sieves with different particle sizes of 2, 5, 10, 20 and 40 mm together from bottom to top and placing them on the shaking table for coarse screening analysis; (2) stacking sieves with particle sizes of 0.075, 0.25, 0.5, 1 and 2 mm from bottom to top and placing them on the shaking table again for fine screening of the remaining soil sample; (3) weighing the remaining soil in each sieve and recording its percentage in the total mass. The experiment results are shown in Table 1 and Table 2.

It can be seen from Table 1 and Table 2 that fine content (less than 0.075 mm) in the soil sample was about 75.9%; sand (0.075~2 mm) accounted for 11.6% and gravel (2~10 mm) was 7.4%. According to the Soil Engineering Classification Criterion (GBJ145-90) of China [29], the high liquid limit soil in Liyu highway could be classified as fine soil.

#### 3.1.2. Chemical Composition Analysis

The objective of the chemical composition analysis test was to determine the content and proportion of oxides of some elements in the soil sample, so as to distinguish clay minerals. In this test, 200 g of soil sample was weighed and a series of tests were carried out, including pH test, ignition loss test and soluble salt determination test, according to the Soil Engineering Classification Criterion (GBJ145-90) of China [29]. The test results are listed in Table 3.

Table 3 shows that the content of SiO_2_ in the soil sample was the highest, more than half of all chemical components and reaching up to 56.19%. The content of Al_2_O_3_ exceeded one-third, accounting for 33.58%. As well accepted, the mineral compositions were normally judged by the silicon–aluminum molecular ratio *S_a_*. The relationship between mineral compositions and *S_a_* can be seen in Table 4.

Therefore, the silicon–aluminum molecular ratio *S_a_* of the soil sample in this test can be calculated as follows:

SiO_2_ molecular ratio = SiO_2_%/SiO_2_ molecular weight = 56.19%/60 = 0.0094 (1)

Al_2_O_3_ molecular ratio = Al_2_O_3_%/Al_2_O_3_ molecular weight = 33.58%/102 = 0.0032 (2)

*S_a_* = SiO_2_ molecular ratio/Al_2_O_3_ molecular ratio = 0.0094/0.0032 = 2.94 (3)

Comparing the results in Table 4, it can be seen that the silicon–aluminum molecular ratio *S_a_* of the soil sample was between the values of illite and kaolinite. Thus, it could be concluded that the soil sample had illite and kaolinite. Because both of them have the characteristics of water swelling, it could be considered that the high liquid limit soil in Liyu highway has poor water stability and significant water swelling properties.

#### 3.1.3. Limit Moisture Content Test

Moisture content is an important index affecting the state of soil. It is of great significance to determine the liquid limit and plastic limit of soil in Liyu highway. In this test, the soil sample was firstly dried, and then the moisture contents of samples were controlled near the liquid limit, near the plastic limit and between them. The cone penetration *h* and corresponding moisture content ω of soil samples were measured by using a liquid–plastic combine tester with a 76 g cone.

The test results reported the relationship between cone penetration and moisture content, having the following calculation formula:

*h* = 0.5653*ω* − 14.4 (4)

**Figure 2:**
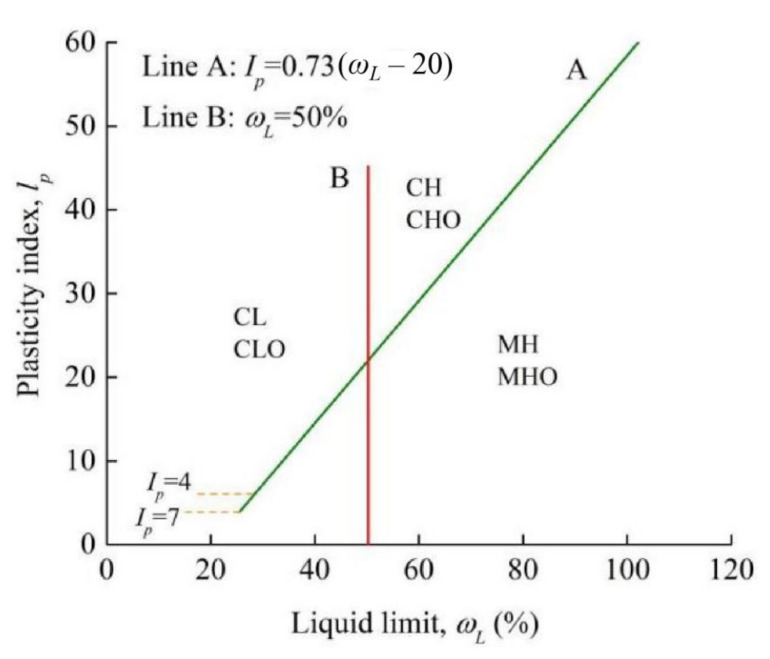
Plasticity chart.

Based on Equation (4), it could be found that the cone penetrations *h* = 2 mm corresponded to the moisture content of *ω* = 29.0%, and cone penetrations *h* = 17 mm corresponded to moisture content of *ω* = 55.6%. Therefore, the liquid limit, plastic limit and plasticity index of the soil sample could be determined as 55.6%, 29.0% and 26.6%, respectively. According to the plasticity chart (Figure 2) of Specifications for Design of Highway Subgrades (JTG-D30-2015) of China [30], the soils in Liyu highway could be grouped into the high liquid limit clay, with the code CH.

### 3.2. Mechanical Properties of High Liquid Limit Soil

#### 3.2.1. Compaction Test

**Figure 3:**
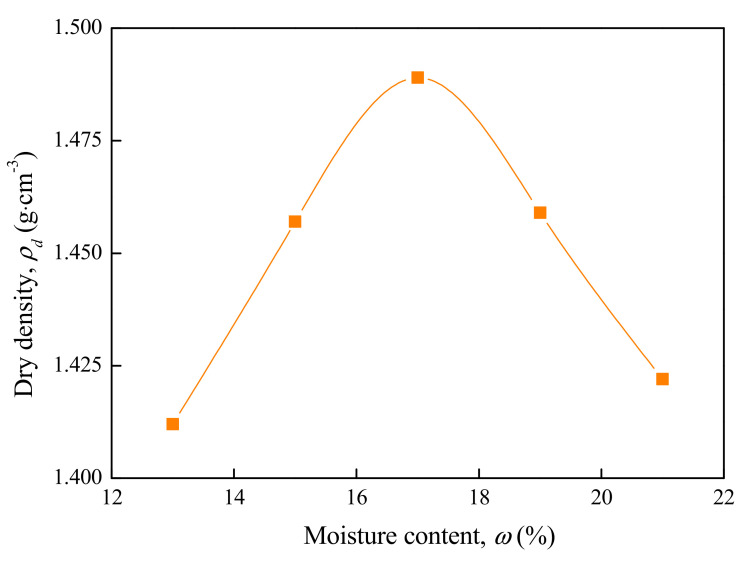
Relationship between dry density and moisture content.

A compaction test is often used to determine the maximum dry density and optimal moisture content of soil. Particle analysis showed that there were some particles in the soil sample with a size larger than 20 mm. According to the Test Method of Soils for Highway Engineering (JTG3430-2020) of China [28], the method of heavy compaction should be adopted in this compaction test. The compaction test results of soil samples with moisture contents of 13%, 15%, 17%, 19%, 21% and natural moisture content are shown in Table 5 and Figure 3.

It can be seen from Figure 3 that for the high liquid limit soils in Liyu highway, the dry density increased with the moisture content before reaching the peak value. After that, it gradually decreased as the moisture content increased. The optimal moisture content of high liquid limit soils was *ω* = 17.2%, and the maximum dry density was *ρ* = 1.49 g·cm^−3^. It was found that the optimal moisture content was at a relatively high level due to the soil containing many hydrophilic minerals. In addition, under the condition of natural moisture content, the dry density of high liquid limit soil was small, which easily leads to a low degree of compaction.

#### 3.2.2. Shear Test

**Figure 4:**
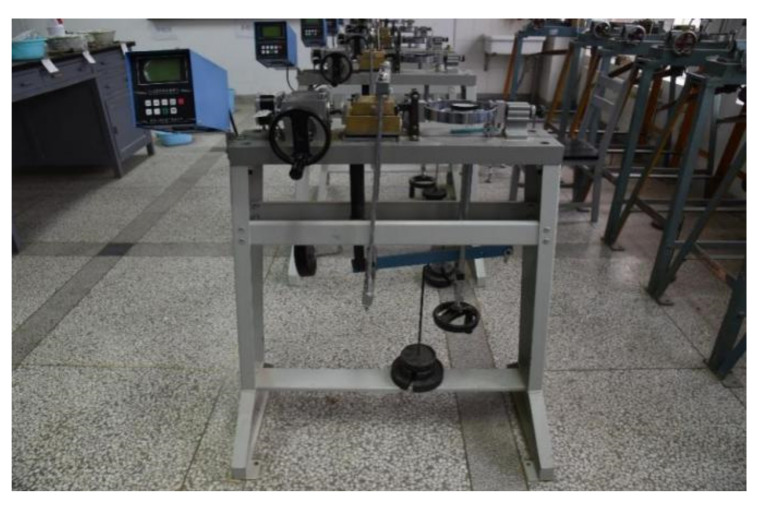
Shear test system.

**Figure 5:**
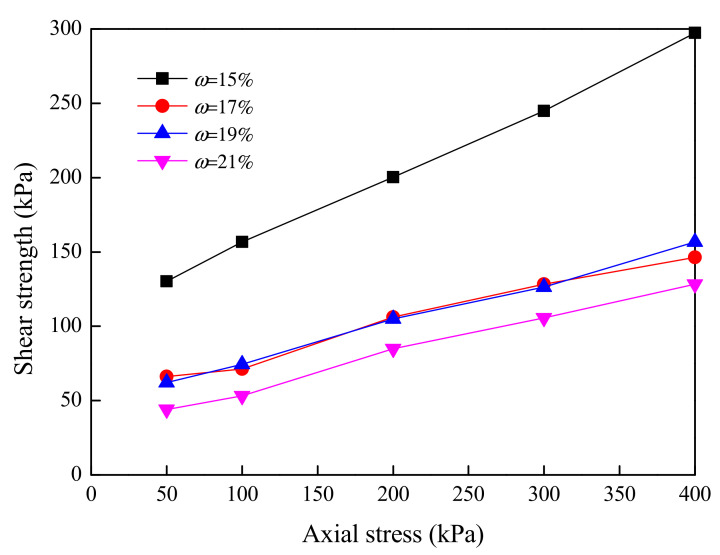
Relationship between shear strength and axial load under the conditions of different moisture contents.

**Figure 6:**
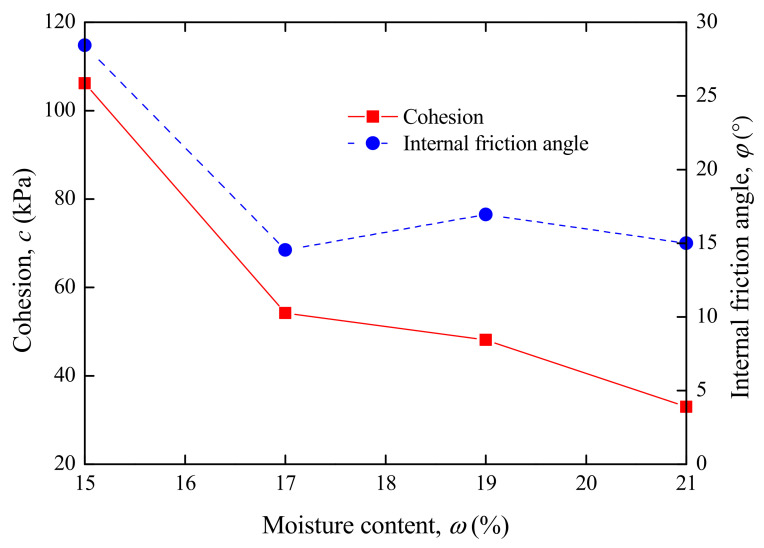
Relationship between cohesion (internal friction angle) and moisture content.

The development laws of shear strength, cohesion c and internal friction angle φ of high liquid limit soil in Liyu highway with the moisture content were investigated. The volume of tested soil samples was 200 mm^3^ with the diameter of 70 mm and the height of 52 mm. Soil samples were prepared with the moisture contents of *ω* = 15%, 17%, 19% and 21%. The axial loads acting on the samples were 50, 100, 200, 300 and 400 kPa during the shearing test. The shear test system is illustrated in Figure 4. The results of shear strength are provided in Figure 5, and results of cohesion and internal friction angle are shown in Figure 6.

It can be found from Figure 5 that the moisture content of high liquid limit soil had a significant influence on the shear strength. In general, the shear strength decreased with the moisture content under the same condition of axial load. When the moisture content did not exceed its optimal value, the shear strength decreased rapidly with the moisture content. However, the decrease in shear strength was not significant when the moisture content was larger than the optimal value.

The results in Figure 6 show that the water had an obvious weakening effect on the cohesion and internal friction angle of high liquid limit soils. Like the shear strength, the cohesion and internal friction angle presented a sharp decrease as the moisture content increased (less than the optimal value). When the moisture content exceeded this value, both parameters entered a slight decrease stage.

#### 3.2.3. Bearing Ratio Test

California bearing ratio (CBR) is an important index for the bearing capacity of highway subgrade, estimating whether soils can be used as the filling materials in subgrade. Table 6 presents the requirements for the minimum CBR for filling materials of subgrade at different depths according to the Specifications for Design of Highway Subgrades (JTG-D30-2015) of China [30].

In this bearing ratio test, four group samples with the diameter of 15.2 cm and height of 12 cm were prepared, according to the optimal moisture content of high liquid limit soil. The quality of each sample could be determined using the following equation:

(5) $$G=2177K\rho_{d\max}\cdot \left(1+\omega_{opt}\right)$$

where *G* represents the sample quality in grams; 2177 is the volume in cm^3^; *ρ_d_*_max_ denotes the maximum dry density in g·cm^−3^; *ω_opt_* is the optimal moisture content in %; and *K* represents the sample compaction degree, being equal to 93%, 94%, 96% and 100% in this test.

**Figure 7:**
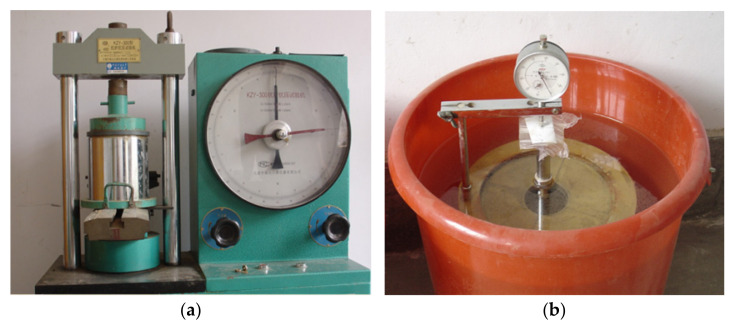
Soil sample dilation experiment. (**a**) Preloading of sample. (**b**) Water absorption.

**Figure 8:**
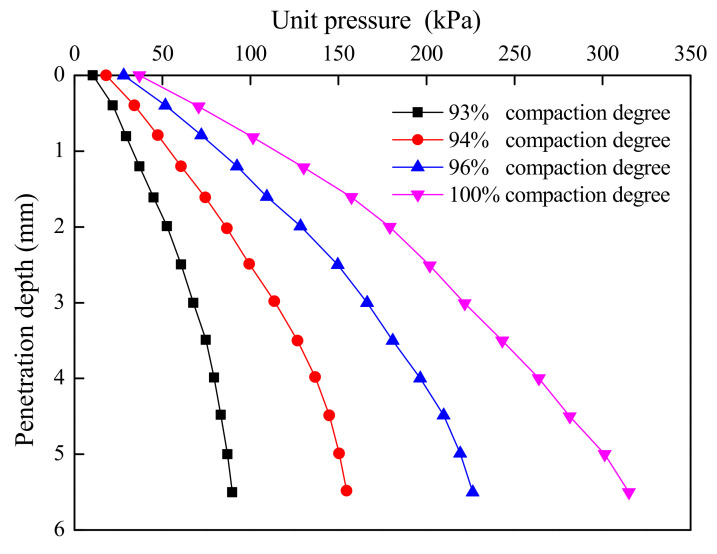
Penetration test results.

The soil sample dilation experiment is illustrated in Figure 7. After the water absorption and dilation measurement, the standard penetration test was carried out. The test results are shown in Figure 8 and Table 7. The ratio of unit pressure (with penetration of 2.5 mm) to standard pressure was used as the CBR of the material and was calculated as:

(6) $$CBR=\frac{p}{7000}\times 100$$

where *p* represents the unit pressure in kPa.

It can be found from Table 7 that a greater compaction degree of high liquid limit soil led to a larger expansion rate and higher CBR value. When the compaction degree was 100%, the maximum CBR of plain high liquid limit soil was only 2.95. As listed in Table 6, the minimum CBR of filling materials in the highway was 3. Therefore, the untreated high liquid limit soils could not be directly utilized as the filling materials in subgrade in Liyu highway, from the aspect of CBR value.

#### 3.2.4. Resilience Modulus Test

Resilience modulus reflects the ability of deformation recovery of subgrade under instantaneous load. It is defined as the ratio of material stress to the corresponding resilience strain. Resilience modulus is mainly affected by the filling material type, compaction degree and moisture content. The influences of compaction degree and moisture content on the resilience modulus of high liquid limit soil were tested. The resilience modulus can be calculated by the following equation:

(7) $$E_{r}=\frac{\pi PD}{4L}\left(1-\mu^{2}\right)$$

where *P* represents the unit pressure in MPa, *D* is the radius of the bearing plate in mm, *L* denotes the resilience deformation corresponding to the unit pressure in mm and *μ* is Poisson’s ratio and is equal to 0.25.

Table 8 and Table 9 show the development laws of resilience modulus of high liquid limit soils with moisture content or compaction degree. The moisture content had a significant influence on the resilience modulus of high liquid limit soil. When the compaction degree was 96%, its maximum resilience modulus could be achieved with a moisture content of 17%. This indicated that under the same compaction degree, the optimal moisture content would lead to the maximum resilience modulus of high liquid limit soil. Table 9 shows that when the moisture content was kept constant, a greater compaction degree caused a larger resilience modulus. The maximum resilience modulus of high liquid limit soil was 26.3 MPa with the compaction degree of 96%. According to the Specifications for Design of Highway Asphalt Pavement (JTGD50-2017) of China [31], the maximum resilience modulus of high liquid limit soil was lower than the required minimum resilience modulus (40 MPa) for upper roadbed. From the aspect of resilience modulus, the high liquid limit soil in Liyu highway could not be directly used as the filling materials in subgrade as well.

## 4. Mechanical Properties of Tunnel-Slag-Improved High Liquid Limit Soil

### 4.1. Mechanical Properties of Tunnel Slag

**Figure 9:**
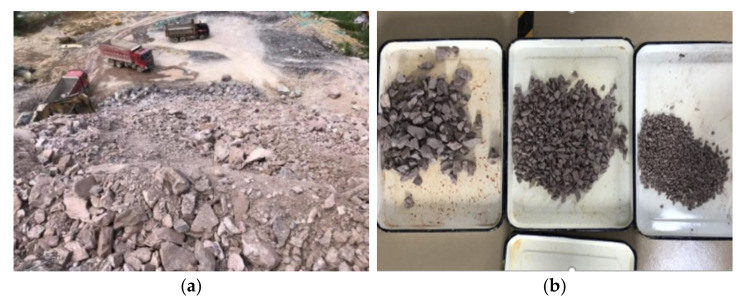
Tunnel slag taken from Wenwei tunnel. (**a**) Original rocks after blasting. (**b**) Tunnel slag used in the experiment.

As shown in Figure 9a, rocks were taken from Wenwei tunnel of Liyu highway, and they were mainly composed of completely weathered gneiss. The original rocks processed through coarse crushing, medium crushing, fine crushing, dust removal and cleaning led to the tunnel slag used in subgrade, as illustrated in Figure 9b.

The physical properties of tunnel slag are presented in Table 10. The tunnel slag had a rough surface and uniform distribution of particle size, which were beneficial to its application in improving the properties of the high liquid limit soils.

### 4.2. Mechanical Properties of Tunnel-Slag-Improved High Liquid Limit Soil

#### 4.2.1. Compaction Test

**Figure 10:**
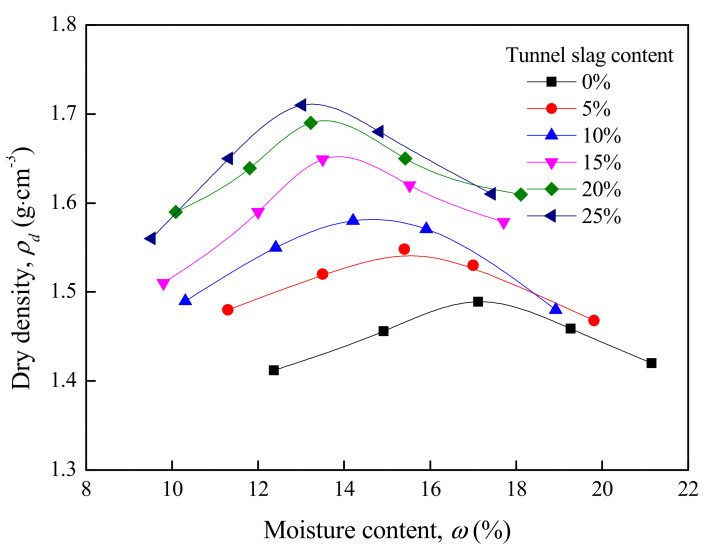
Relationship between dry density and moisture content of tunnel-slag-improved high liquid limit soil.

The compaction test of tunnel-slag-improved high liquid limit soil was conducted. Like the method described in Section 3.2.1, the soil samples with different moisture contents were firstly prepared, and then the tunnel slag in different contents was added to soil samples. The tunnel slag contents in this test were 5%, 10%, 15%, 20% and 25%. The test results are shown in Figure 10.

It can be seen from Figure 10 and Table 11 that the maximum dry density of improved high liquid limit soil showed an increasing trend with the tunnel slag content. This was because tunnel slag in soils acted as the coarse particles and the fine particles tightly filled around it, leading to a significant increase in density of improved soils. Furthermore, the optimal moisture content decreased as the tunnel slag content increased. This could be explained by the addition of tunnel slag causing the decrease in the contents of fine and clay particles in unit soil. That is, the hydrophilic mineral composition in soils decreased with the increase in tunnel slag content.

#### 4.2.2. Shear Test

**Figure 11:**
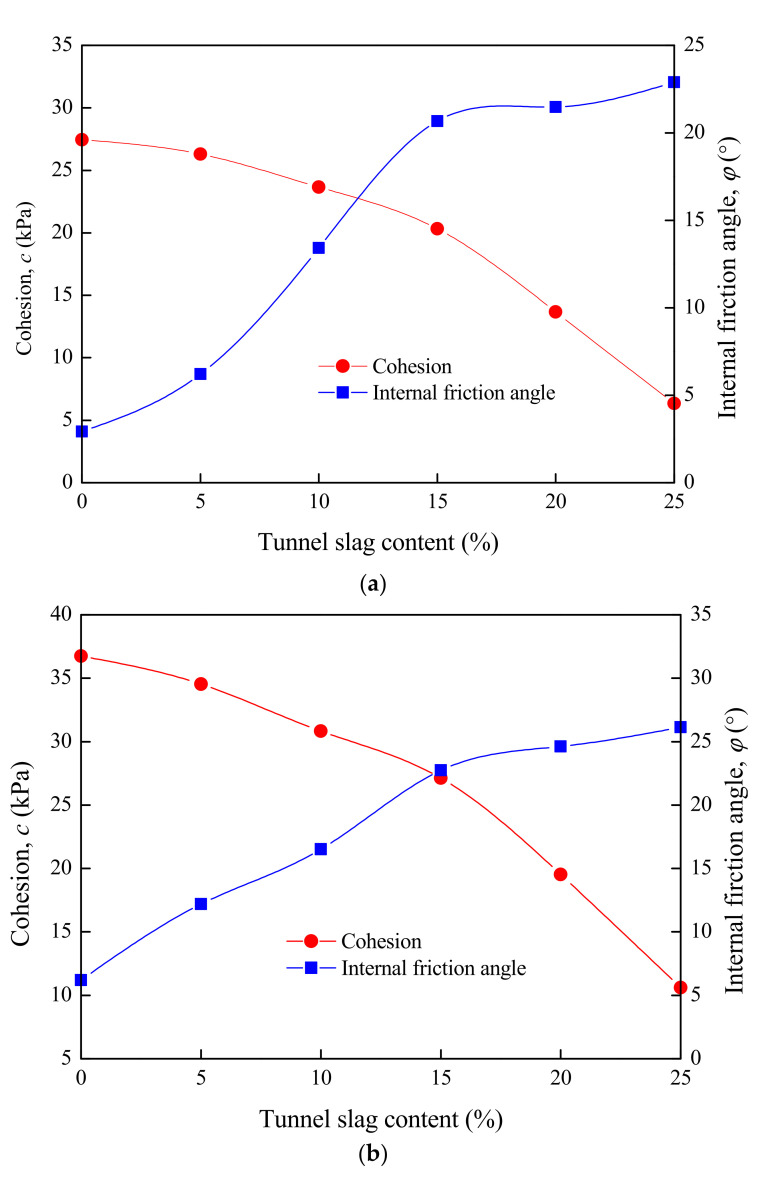

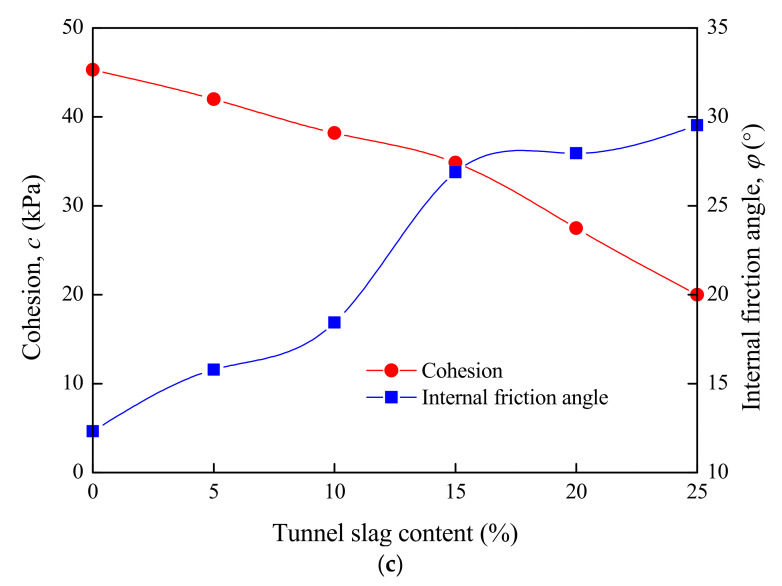
Cohesion and internal friction angle of tunnel-slag-improved high liquid limit soil. (**a**) Cohesion and internal friction angle under the condition of compaction degree of 93%. (**b**) Cohesion and internal friction angle under the condition of compaction degree of 94%. (**c**) Cohesion and internal friction angle under the condition of compaction degree of 96%.

The cohesion and internal friction angle of tunnel-slag-improved high liquid limit soils were tested. Similarly, the scheme of tunnel slag contents of 5%, 10%, 15%, 20% and 25% was adopted. The soil samples for the shear test were prepared with the optimal moisture content and maximum dry density (obtained in Section 4.2.1). In addition, the compaction degrees of 93%, 94% and 96% were taken. Considering the tunnel slag in soils, the GDS large-scale direct shear apparatus was used to perform the shear test of improved soil samples, with the sample scales of 300 mm × 300 mm × 150 mm in dimension. The results are shown in Figure 11.

Figure 11 shows the development laws of cohesion and internal friction angle of improved soils with tunnel slag contents and compaction degrees. The cohesion and internal friction angle increased with the compaction degree under the condition of the same tunnel slag content. The cohesion of improved high liquid limit soils presented a decreasing trend as the tunnel slag content increased, but the internal friction angle increased with the content. Taking the results in Figure 11a as an example, when the tunnel slag content was less than 15%, the decrease rate of cohesion was at a relatively low level. Instead, the internal friction angle showed a rapid increase. If the tunnel slag content was greater than 15%, the cohesion decreased rapidly, but the internal friction angle increased slowly. This conclusion was also validated by the results in Figure 11b,c.

The research found that in the improved high liquid limit soils, tunnel slag formed the soil skeleton, which mainly provided friction without cohesion. The fine particles majorly contributed to the cohesion of soils. When tunnel slag content increased, the content of fine particles continued to decrease, leading to an increase in internal friction angle and a decrease in cohesion. It was supposed that there should be a reasonable threshold value *η* for tunnel slag content in high liquid limit soils. If the tunnel slag content was less than *η*, the shear strength of improved soils was dominated by fine particles. The cohesion was basically unchanged and the internal friction angle had a significant increase with the tunnel slag content. This would cause an increase in the shear strength of improved high liquid limit soils. Once the tunnel slag content exceeded *η*, it played an increasing role in the shear strength of soils. Although the internal friction angle still increased with tunnel slag content, the incremental value was very limited compared with the decrease in cohesion. Therefore, the shear strength of improved high liquid limit soils generally presented a decreasing trend. Based on the results in Figure 11, the value of 15% could be determined as the optimal tunnel slag content in Liyu highway.

#### 4.2.3. Bearing Ratio Test

**Figure 12:**
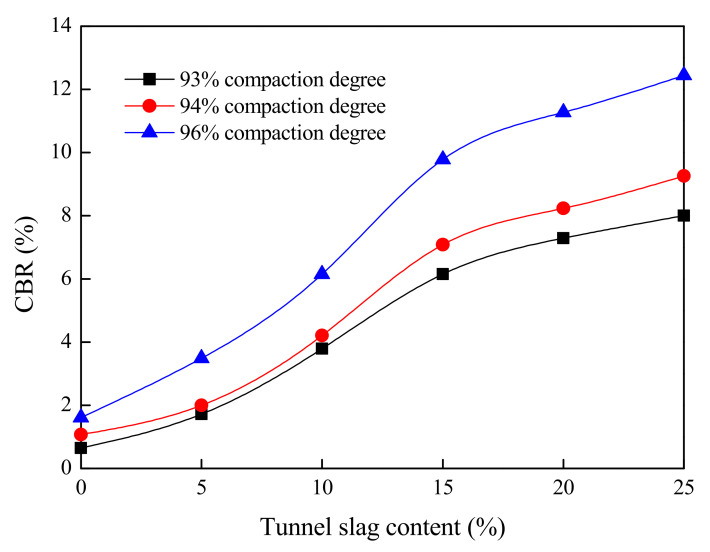
Relationship between CBR and tunnel slag.

**Figure 13:**
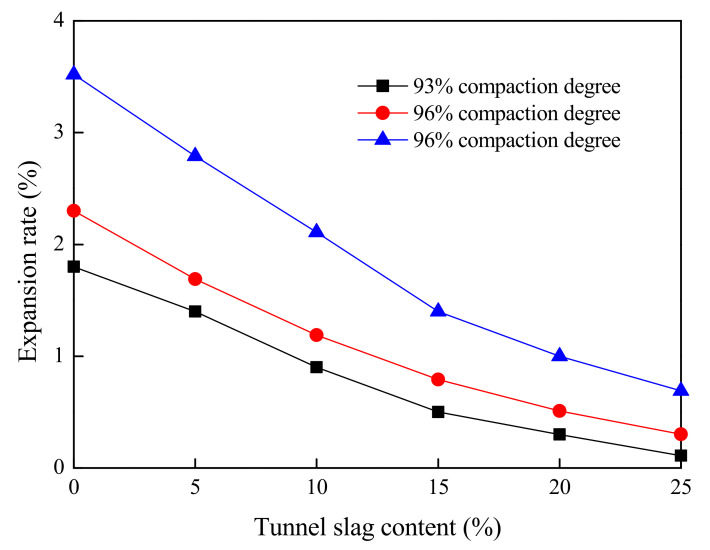
Relationship between expansion rate and tunnel slag.

This section presents the results of the bearing ratio test of tunnel-slag-improved high liquid limit soils. All parameters in this test were kept the same as those in Section 4.2.2. The test results are shown in Figure 12 and Figure 13.

It can be seen from Figure 12 and Figure 13 that a higher CBR and a smaller expansion rate could be obtained as tunnel slag content increased. This was because the coarse particle content in untreated high liquid limit soils was very low, being dispersed in soils. The skeleton effect of soils was not obvious before adding the tunnel slag, and the soil integrity was poor and its bearing capacity was low. The spatial structure of high liquid limit soils was rearranged by tunnel slag mixing; the tunnel slag could act as the soil skeleton and fine particles filled around these coarse particles, leading to a dense gravel–soil structure and a great enhancement of integrity of the soil. Furthermore, it can be seen from Figure 12 and Figure 13 that when the content of tunnel slag was less than 15%, the CBR greatly increased and the expansion rate obviously decreased with the content. However, if this content exceeded 15%, the CBR of improved soils changed into a slow increase trend and the expansion rate slightly decreased. This indicated that the further addition of tunnel slag in soils would not exert a significant influence on both parameters.

According to the Specifications for Design of Highway Subgrades (JTG-D30-2015) of China [30], the improved high liquid limit soils with tunnel slag content of 10% could meet the minimum strength requirement for lower embankment under the condition of compaction degree of 93%. The improved soils with tunnel slag content of 10% could meet the minimum strength requirement for upper embankment under the condition of compaction degree of 94%. However, a sufficient safety reserve could not be achieved under this condition. The improved soils with tunnel slag content of 15% could meet the minimum strength requirement for both the lower and upper roadbeds under the condition of compaction degree of 96%, simultaneously showing sufficient reserve.

## 5. Numerical Modeling

### 5.1. Model and Parameters

**Figure 14:**
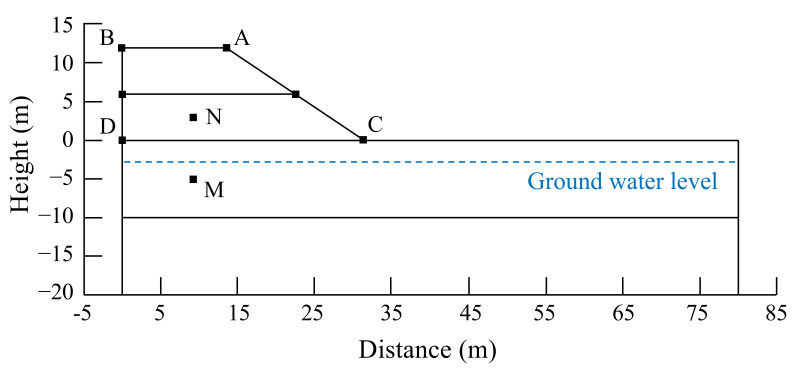
Numerical model.

In order to further validate the feasibility of tunnel-slag-improved high liquid limit soils in subgrade in Liyu highway, numerical simulation focusing on the stability of subgrade slope and long-term settlements of subgrade was conducted. The GeoStudio2018 software was used in the numerical calculation. A test section of Liyu highway was selected for analysis. A half model was used in the calculation due to symmetry, as shown in Figure 14. The subgrade had a filling height of 12 m, a top width of 27 m and a slope gradient of 1:1.5, and the width of the foundation was 80 m. According to the geological investigation, groundwater was located 3 m below the surface. The soil strata could be divided into subclay layer, residual clay layer, strongly weathered silty sandstone layer and weakly weathered silty sandstone layer from the surface to bottom. The weakly weathered silty sandstone layer was located 20 m below the surface. The influence of subgrade filling on its deformation could be neglected. The points of A, B, C, D, M and N were chosen as the monitoring ones and had the coordinates of (13.5, 12), (0, 12), (31.5, 0), (0, 0), (9, −5) and (9, 3), respectively. The bottom of the numerical model was totally fixed. Its left and right sides were horizontally fixed and could generate vertical displacements. It was assumed that the subgrade only drained on the slope surface and the drainage boundary pressure was always equal to atmospheric pressure.

Based on the geological investigation and experimental tests, the physical and mechanical parameters of soil layers are shown in Table 12. The linear elastic model was employed to describe the mechanical behaviors of foundation soils in the numerical simulation, considering their small deformations during the construction and operation process. The Mohr–Coulomb elastoplastic model was applied to simulate the deformation of tunnel-slag-improved high liquid limit soils. According to the test results in Section 4, the cohesion and internal friction angle of improved high liquid limit soils at the tunnel slag content of 15% were 28° and 35 kPa, respectively. The Young’s modulus of the filling materials of subgrade could be determined, according to the Technical Specifications for Construction of Highway Subgrades (JGT/T 3610-2019) of China [32], as follows:

(8) $$E=k_{b}p_{a}{\left(\frac{\sigma }{p_{a}}\right)}^{n}$$

where *k_b_* and *n* are the fitting parameters, *p_a_* is the standard atmospheric pressure and *σ* is the average pressure. According to the results of the triaxial consolidated undrained test, *k_b_* and *n* were 70 and 0.72, respectively. The vehicle load and pavement load of Liyu highway were equal to 10.5 kPa and 17.5 kPa, respectively. Thus, the total load on subgrade *σ* = 28 kPa.

Soils below the groundwater level were saturated, and their permeability coefficients remained unchanged. The permeability coefficients of saturated residual clay and strongly weathered silty sandstone were 6.0 × 10^−5^ cm/s and 7.2 × 10^−4^ cm/s, respectively. Soils above the groundwater level were unsaturated, and the permeability coefficients of improved high liquid limit soil and subclay were 4.0 × 10^−7^ cm/s and 4.2 × 10^−6^ cm/s, respectively.

### 5.2. Subgrade Filling Process

According to the Technical Specifications for Construction of Highway Subgrades (JGT/T 3610-2019) of China [32], the thickness of each layer of filling soils in subgrade should not exceed 30 cm. The work of two layers of filling soils could be completed every day on Liyu highway. The work should allow sufficient time to consolidate and stabilize the soils after filling due to the characteristics of small permeability coefficient and slow dissipation of pore water pressure of high liquid limit soils. Therefore, the whole subgrade filling process for Liyu highway was divided into two stages. The first filling stage spanned 10 days, meaning that the height of 6 m of filling soils was completed. Subsequently, the consolidation lasted 30 days. The second stage also lasted 10 days and the subgrade filling was finally completed. The construction period of pavement works was controlled within 6 months, which meant the highway officially opened half a year after the subgrade completion.

### 5.3. Subgrade Slope Stability of Tunnel-Slag-Improved High Liquid Limit Soil

**Figure 15:**
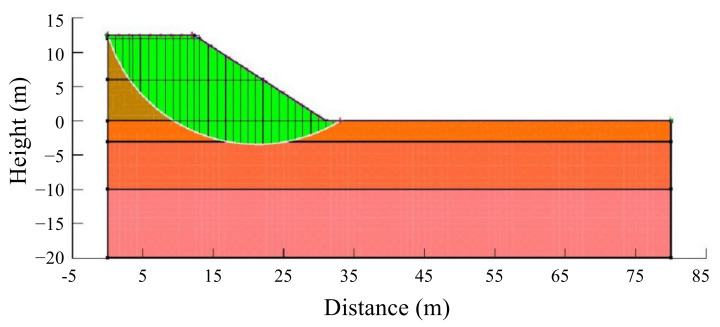
Diagram of critical failure state of subgrade slope.

Figure 15 shows the critical failure state of subgrade slope of tunnel-slag-improved high liquid limit soil in Liyu highway. Table 13 lists the safety factors of subgrade slope stability using different analysis methods. The minimum safety factor was achieved using the Janbu method. All safety factors of subgrade slope stability calculated using these four methods were larger than 1. This demonstrated that the subgrade slope stability of improved high liquid limit soil at tunnel slag content of 15% could meet the specification requirements [30].

### 5.4. Subgrade Settlement of Tunnel-Slag-Improved High Liquid Limit Soil

**Figure 16:**
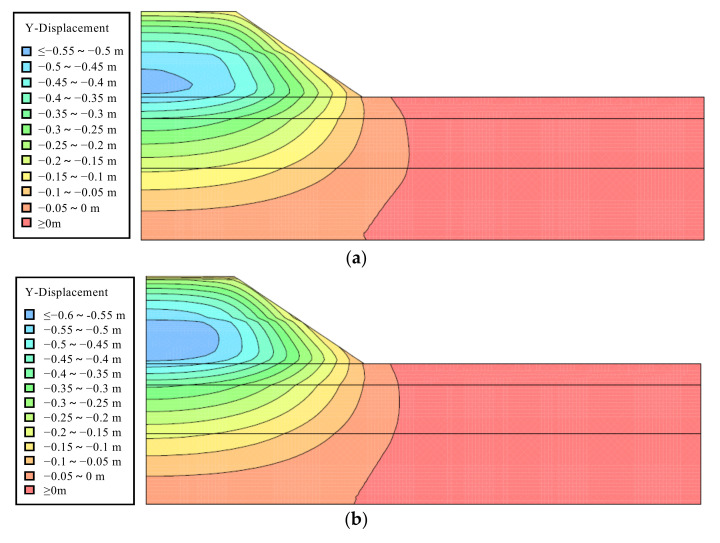

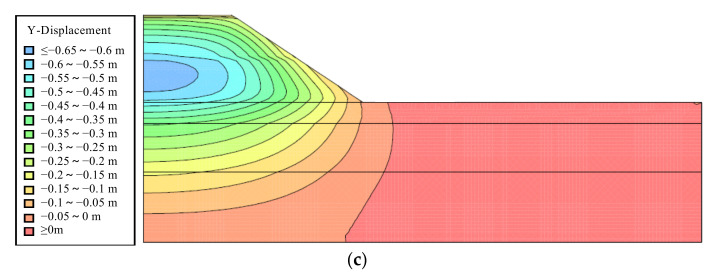
Displacement nephograms at different times. (**a**) Displacement nephogram at the end of consolidation. (**b**) Displacement nephogram after one-year operation. (**c**) Displacement nephogram after two-year operation.

Figure 16 illustrates the displacement nephograms of subgrade at the end of the consolidation period, after one-year operation and after two-year operation. Through the displacement nephograms, it could be found that the subgrade displacement was almost completed during the consolidation period. A very small displacement of subgrade occurred during the operation period. In addition, the level of uneven displacement of subgrade surface was very low. During the consolidation period, the settlement of point B was approximately 10 cm. This indicated that sufficient deformation should be reserved and the subgrade was required to be filled to reach the height of 12.10 m.

**Figure 17:**
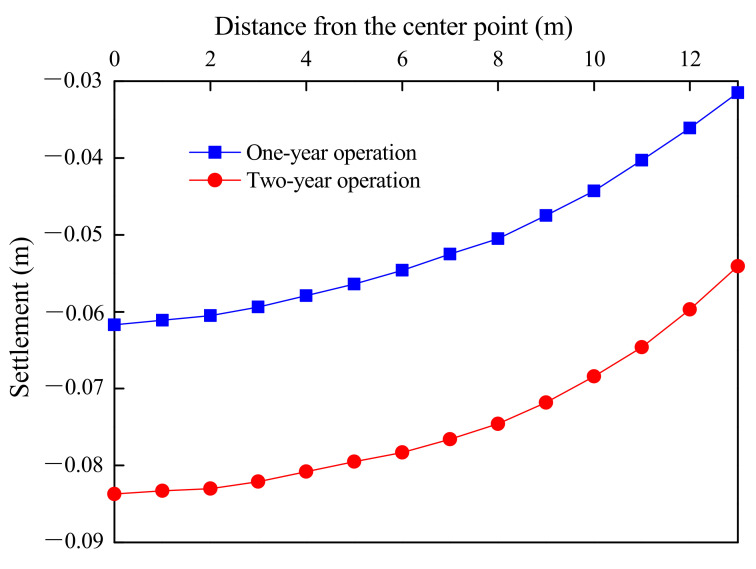
Subgrade surface settlements after one- and two-year operations.

Furthermore, Figure 17 shows the subgrade surface settlements after one- and two-year operations. The maximum uneven settlements of subgrade surface after one- and two-year operations could both be controlled within 3 cm, which met the specification requirements [32]. Therefore, based on the numerical results, it was safe to conclude that it was feasible to utilize of tunnel-slag-improved high liquid limit soils as filling materials of subgrade in Liyu highway.

## 6. Application of Tunnel-Slag-Improved High Liquid Limit Soil in Subgrade in Liyu Tunnel

**Figure 18:**
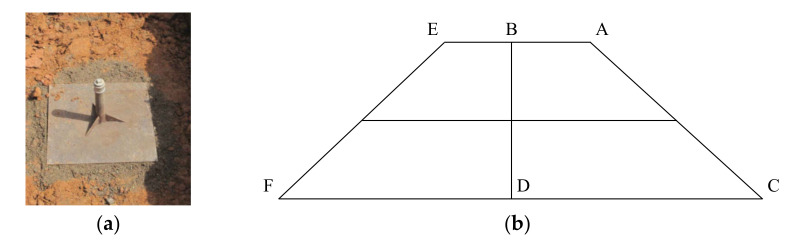
Illustrnd arrangement of settlement plates. (**a**) Settlement plate. (**b**) Layout of monitoring points.

The improved high liquid limit soil at tunnel slag content of 15% was applied as the filling materials in subgrade in Liyu highway. The test section of a total length of 100 m was selected, from the starting point of K58 + 300 to the ending point of K58 + 400. Dynamic monitoring of the improved soil subgrade settlements was conducted. The arrangement positions of settlement plates used in the dynamic monitoring scheme are shown in Figure 18. According to the settlement control standard for highway subgrade, the settlement rate of the middle surface should be less than 10 mm/day for the subgrade to be considered to be in a stable state. If the monthly average settlement rate of the middle surface is less than 10 mm/month, the subbase construction of subgrade can be carried out. As shown in Figure 18, six settlement plates were arranged and the settlements during 230 days were monitored from the subgrade construction to 180 days after construction was completed. The monitoring data are listed in Table 14. Table 15 exhibits the comparison of final subgrade settlements between numerical results and field monitoring data.

It can be seen from Table 14 that in Liyu highway, the maximum subgrade settlement occurred in point D, and its early settlement rate reached 20 mm/day. However, the subgrade settlement tended to be stable during the consolidation period. The monthly average settlement rate of the middle surface was less than 10 mm/month. Furthermore, as shown in Table 15, the difference of subgrade settlements between numerical results and monitoring data could be controlled within 5%, which well validated the effectiveness of numerical simulation. Of course, it also demonstrated that it was easy and reliable to use numerical analysis to guide the following subgrade construction. In summary, a good application of tunnel-slag-improved high liquid limit soil in subgrade in Liyu highway was achieved.

## 7. Conclusions

This study investigated the physical and mechanical properties of plain and tunnel-slag-improved high liquid limit soils, based on the Liyu highway, Guangxi Province, China. The feasibility of the utilization of tunnel-slag-improved high liquid limit soil as filling material of subgrade was analyzed using a numerical method. The field application and monitoring of improved soil in subgrade practice were carried out. The main findings can be summarized as follows:

(1) The shear strength parameters of plain high liquid limit soil in Liyu highway decreased as the moisture content increased. The CBR value of plain soil under the conditions of maximum dry density and compaction degree of 100% was 2.95, which was smaller than the minimum CBR value for filling materials in highway subgrade. In addition, its maximum resilience modulus was 26.3 MPa, also far less than the required minimum value of filling materials. It was unacceptable to directly utilize the untreated plain high liquid limit soil in subgrade practice.

(2) The optimal moisture content of the improved high liquid limit soil showed a decreasing trend with tunnel slag content due to the decrease in hydrophilic mineral composition in soils after tunnel slag addition. The mixed tunnel slag having a higher density than soil particles formed the soil skeleton, only providing friction without cohesion. The maximum dry density and internal friction angle of the improved soil increased as tunnel slag content increased, but its cohesion exhibited an opposite trend. A higher CBR value and a smaller expansion rate were observed with increased tunnel slag content, indicating a significant enhancement of integrity of improved high liquid limit soil. An appropriate content of tunnel slag could positively affect the properties of high liquid limit soil. In this engineering practice, the value of 15% was the optimal tunnel slag content, and the performance of improved high liquid limit soil at this tunnel slag content could meet the specification requirements.

(3) Numerical results showed that the stability of subgrade slope using filling materials of tunnel-slag-improved high liquid limit soil could be well guaranteed. The subgrade settlement was also within the range allowed in specifications. A good application of tunnel-slag-improved high liquid limit soil as subgrade filling materials in Liyu highway was achieved.

## Figures and Tables

**Table 1:** Coarse screening analysis data.

Pore Size (mm)	Accumulated Quality of Remaining Soil (g)	Quality of Filtered Soil (g)	Mass Percentage of Filtered Soil to Soil Sample (%)
40	0	2000	100
20	22.1	1977.9	98.9
10	102.2	1897.8	94.9
5	162.7	1837.3	91.9
2	249.6	1750.4	87.5

**Table 2:** Fine screening analysis data.

Pore Size (mm)	Accumulated Quality of Remaining Soil (g)	Quality of Filtered Soil (g)	Mass Percentage of Filtered Soil to Fine Screening Soil Sample (%)	Mass Percentage of Filtered Soil to Soil Sample (%)
2	249.6	1750.4	100	87.5
1	299.8	1700.2	97.1	85.0
0.5	362.9	1637.1	93.5	81.9
0.25	433.5	1566.5	89.5	78.3
0.075	482.4	1517.6	86.7	75.9

**Table 3:** Analysis of mineral composition and chemical components.

Mineral Composition	SiO_2_	Fe_3_O_4_	Al_2_O_3_	CaO	Ca^2+^	Mg^2+^	Cl^−^	SO_4_^2−^
Percentage (%)	56.19	7.02	33.58	3.30	0.002	0.0012	0.0617	0.05

**Table 4:** Relationship between mineral composition and *S_a_*.

Mineral Composition	Montmorillonite	Illite	Kaolinite	Halloysite
Silicon–aluminum molecular ratio Sa	>4	3.0–3.2	2.0–2.2	2.0–2.2

**Table 5:** Compaction test results.

Number	1	2	3	4	5	6
Expected moisture content, *ω_e_* (%)	13	15	17	19	21	Natural moisture content
Density, *ρ* (g·cm^−3^)	1.58	1.67	1.74	1.77	1.76	1.77
Moisture content, *ω* (%)	12.4	14.3	17.1	19.3	21.1	30.3
Dry density, *ρ_d_* (g·cm^−3^)	1.41	1.46	1.49	1.48	1.45	1.36

**Table 6:** Minimum CBR requirements for filling materials of subgrade.

Subgrade Name	Depth below Pavement Bottom (cm)	Minimum CBR of Filling Material (%)	
		Highway, First-Class Road	Second-Class Road
Upper roadbed	0~30	8	6
Lower roadbed	30~80	5	4
Upper embankment	80~150	4	3
Lower embankment	Larger than 150	3	2

**Table 7:** Test results for sample CBR.

Compaction Degree (%)	Dry Density before Water Absorption (g·cm^−3^)	Dry Density after Water Absorption (g·cm^−3^)	Expansion Rate (%)	CBR (%)	Pore Size (mm)
93	1.39	1.38	1.80	0.66	40
94	1.40	1.39	2.30	1.08	20
96	1.43	1.40	3.50	1.63	10
100	1.49	1.45	5.80	2.95	5

**Table 8:** Resilience moduli under the conditions of different moisture contents.

Number	Compaction Degree (%)	Moisture Content (%)	Resilience Modulus (MPa)
1	96	15.2	20.5
2		16.3	22.1
3		17.0	24.2
4		18.2	21.6
5		19.5	18.4

**Table 9:** Resilience moduli under the conditions of different compaction degrees.

Number	Moisture Content (%)	Compaction Degree (%)	Resilience Modulus (MPa)
1	17.2	93	20.1
2		94	22.4
3		96	26.3

**Table 10:** Physical properties of tunnel slag.

Density (g·cm^−3^)	Apparent Density (g·cm^−3^)	Crushing Index (%)	Particle Size, *d* (mm)		
			5 ≤ *d* < 10	10 ≤ *d* < 20	20 ≤ *d* < 40
1.65	2.71	18.5	28%	42%	30%

**Table 11:** The optimal moisture content and maximum dry density of tunnel-slag-improved high liquid limit soil.

Number	Tunnel Slag Content (%)	Optimal Moisture Content (%)	Maximum Dry Density (g·cm^−3^)
1	0	17.2	1.49
2	5	15.4	1.55
3	10	14.2	1.58
4	15	13.5	1.65
5	20	13.2	1.69
6	25	13.0	1.71

**Table 12:** Physical and mechanical parameters of soils obtained from geological survey report and experimental tests.

Group	Bulk Density (kN/m^3^)	Thickness (m)	Young’s Modulus *E* (MPa)	Poisson’s Ratio, *μ*	Cohesion, *c* (kPa)	Internal Friction Angle, *φ* (°)
Improved high liquid limit soil	16.5	12	2.8	0.3	35	28
Subclay	18.6	3	2.7	0.33	19.3	23
Residual clay	19.2	7	6.6	0.32	22	27
Strongly weathered silty sandstone	20.1	10	9.4	0.31	15.1	35

**Table 13:** Safety factors of subgrade slope.

Analysis Method	Swedish Arc Method	Bishop Slice Method	Janbu Method	Morgenstern–Price Method
Safety factor	1.457	1.501	1.392	1.548

**Table 14:** Accumulated settlement of the section K58 + 350.

Days	Accumulated Settlement (mm)					
	A	B	E	C	D	F
0	-	-	-	0	0	0
5	-	-	-	10.3	87.1	10.1
10	-	-	-	18.4	184.1	18.0
25	-	-	-	29.3	205.5	28.5
40	-	-	-	31.2	216.2	30.4
45	-	-	-	30.1	298.8	29.4
50	0	0	0	29.4	388.9	28.6
65	17.6	18.8	18.0	37.1	411.3	36.2
80	29.3	31.5	29.9	40.1	426.2	39.1
95	39.1	42.2	39.9	42.0	438.5	40.9
110	47.6	51.6	48.6	43.3	449.0	42.3
125	55.0	60.0	56.2	44.2	458.1	43.2
140	61.5	67.4	62.8	44.8	467.8	43.8
155	67.0	74.0	68.5	45.3	472.4	44.2
170	71.8	79.5	73.3	45.6	477.3	44.5
185	76.0	84.2	77.6	45.9	481.0	44.8
200	79.9	88.7	81.6	46.1	484.2	45.0
215	83.6	92.8	85.4	46.2	487.1	45.1
230	87.1	96.7	89.0	46.3	489.6	45.2

“-” indicated no monitoring data.

**Table 15:** Comparison of subgrade settlements between numerical results and monitoring data.

Position	Settlement (mm)		Error	
	Numerical Result	Monitoring Data	Difference (mm)	Percentage (%)
A	84.5	87.1	2.6	3.1
B	98.4	96.7	−1.7	−1.7
C	44.3	46.3	2.0	4.4
D	480.0	489.6	9.6	2.0

## Data Availability

Not applicable.

## List of Notations

*c*	Cohesion
*d*	Particle size
*D*	Radius of bearing plate
*G*	Sample quality
*E*	Young’s modulus
*h*	Cone penetration
*k_b_*	Fitting parameters
*K*	Sample compaction degree
*l_p_*	Plasticity index
*L*	Resilience deformation corresponding to the unit pressure
*n*	Fitting parameters
*p_a_*	Standard atmospheric pressure
*P*	Unit pressure
*S_a_*	Silicon–aluminum molecular ratio
*ω*	Moisture content
*ω_e_*	Expected moisture content
*ω_L_*	Liquid limit
*ω_opt_*	Optimal moisture content
*ρ*	Density
*ρ_d_*	Dry density
*ρ_dmax_*	Maximum dry density
*φ*	Internal friction angle
*μ*	Poisson’s ratio
*σ*	Subgrade load
